# Alkaline decomposition of synthetic jarosite with arsenic

**DOI:** 10.1186/1467-4866-14-2

**Published:** 2013-04-08

**Authors:** Francisco Patiño, Mizraim U Flores, Iván A Reyes, Martín Reyes, Juan Hernández, Isauro Rivera, Julio C Juárez

**Affiliations:** 1 Centro de Investigaciones en Materiales y Metalurgia, Universidad Autónoma del Estado de Hidalgo, Carretera Pachuca-Tulancingo km. 4.5, Pachuca, Hidalgo, C.P. 42184, Mexico

**Keywords:** Alkaline decomposition, Synthetic jarosite with arsenic, Kinetics, Reaction order, Activation energy

## Abstract

The widespread use of jarosite-type compounds to eliminate impurities in the hydrometallurgical industry is due to their capability to incorporate several elements into their structures. Some of these elements are of environmental importance (Pb^2+^, Cr^6+^, As^5+^, Cd^2+^, Hg^2+^). For the present paper, AsO_4_^3-^ was incorporated into the lattice of synthetic jarosite in order to carry out a reactivity study. Alkaline decomposition is characterized by removal of sulfate and potassium ions from the lattice and formation of a gel consisting of iron hydroxides with absorbed arsenate. Decomposition curves show an induction period followed by a conversion period. The induction period is independent of particle size and exponentially decreases with temperature. The conversion period is characterized by formation of a hydroxide halo that surrounds an unreacted jarosite core. During the conversion period in NaOH media for [OH^-^] > 8 × 10^-3^ mol L^-1^, the process showed a reaction order of 1.86, and an apparent activation energy of 60.3 kJ mol^-1^ was obtained. On the other hand, during the conversion period in Ca(OH)_2_ media for [OH^-^] > 1.90 × 10^-2^ mol L^-1^, the reaction order was 1.15, and an apparent activation energy of 74.4 kJ mol^-1^ was obtained. The results are consistent with the spherical particle model with decreasing core and chemical control.

## Introduction

Arsenic has been found in underground waters in several countries around the world at levels that surpass those established by the World Health Organization (10 μg L^-1^) [1]. This issue affects more than 70 countries, where the health of approximately 150 million people is at risk. Of the total, 110 million people live in south and south east Asia [2], and the rest are found in countries such as Argentina, Mexico, Chile, Peru, United States, Brazil and Canada [3,4].

In the last decade, several alternatives have been suggested in order to reduce the concentration of arsenic in potable water: coagulation-flocculation, ion exchange, membrane filtration, precipitation with alum, ozone oxidation, precipitation with iron, lime softening, adsorption with minerals, and activated alumina among others. However, only a few of these have proved to be effective [5]. Arsenic cannot be easily eliminated, but it can be combined with other elements, such as iron, and turned into an insoluble compound that is stable in the environment [6].

Jarosites are used to eliminate iron in the zinc mining industry, and their structure can incorporate impurities, such as As and Pb. Dutrizac et al. [7,8] have studied the incorporation of arsenic in the structure of Na and K jarosites. They achieved incorporation of 4% AsO_4_ in synthetic jarosite and 1% in synthetic natrojarosite. Asta et al. [9] also synthesized jarosites and goethites that, when exposed to acidic pH, showed the capability of absorbing arsenic. Studies on the dissolution of natural and synthetic jarosites not containing arsenic have been carried out at different pH conditions, and it has been found that dissolution is faster at an alkaline pH compared to acidic pH; besides, dissolution occurs in an erratic manner [10,11]. Smith et al. [12] synthesized lead- and lead-arsenic jarosites, which underwent dissolution at pH 2 and 8, but kinetic study of decomposition was not conducted. Savage et al. [13] synthesized jarosites with arsenic, varying the potassium arsenate concentration in order to study the substitution of arsenic for sulfur inside the unit cell; they concluded that arsenate expands the unit cell, since the bond distance between arsenic and oxygen is longer than that between sulfur and oxygen. However, a study on the reactivity of these compounds was not conducted. Patiño et al. [14-18] have thoroughly studied the reactivity of argentian jarosites in alkaline media, where cyanidation was also carried out in order to recover the silver contained in those compounds. Recently, Reyes et al. [19] have synthesized synthetic natrojarosite with arsenic, incorporating 1% AsO_4_ in its structure; they studied the alkaline reactivity in NaOH and Ca(OH)_2_, and they found that the decomposition is strongly related to the reaction conditions, such as temperature, pH and particle size [19].

For this work, a sample of jarosite with arsenic was synthesized, incorporating ≈ 4% AsO_4_ in its structure. Next, a kinetic study of alkaline decomposition was carried out to obtain the apparent activation energy and reaction order, and to compare these data with the results obtained by Reyes et al. [19] in order to determine the differences in the reaction rates between both synthetic jarosites with arsenic.

## Materials and experimental procedure

### Materials

For the alkaline decomposition study, a sample of synthetic jarosite with arsenic was synthesized. The synthesis method was chosen from previous work published by Dutrizac and Cruells [7,8,18]. Synthesis was carried out in a glass kettle with mechanical stirring. The synthesis conditions were the following: 0.3 mol L^-1^ Fe(SO_4_)_3_, 0.2 mol L^-1^ K_2_SO_4_, 0.066 mol L^-1^ KH_2_AsO_4_ and 0.01 mol L^-1^ H_2_SO_4_. The solution was placed in a glass reactor with a spiral condenser coupled on a heating plaque, with automatic temperature control (Super-Nuova/Barnstead-Thermoline) and three-bladed mechanical stirring. The solution was kept at constant temperature of 94°C (367.6K), a stirring rate of 500 min^-1^ for 24 h and 57.41 g of synthetic jarosite with arsenic were obtained. For quantitative elemental analysis it was necessary to first dissolve a sample (1 g) in a 1:1 solution of water and concentrated hydrochloric acid. The solution was kept in a beaker, with magnetic stirring, and heated at 70°C (343K) until no solid products were noticed; then this solution was transferred to a 100 mL volume. Sample dissolution and standards were prepared in identical matrixes. The resulting sample underwent characterization by chemical analysis, X-Ray Diffraction XRD (Siemens D-500) and Scanning Electron Microscopy SEM (JEOL JSM-6300) equipped with Energy-Dispersive X-ray Spectroscopy EDS (Thermo). Particle size was determined with a series of Tyler sieves (USA Standard Testing Sieve, ASTM-11 specifications) with the following mesh diameters: 125, 90, 75, 53, 45, 38 and 25 μm. Characterization confirmed a simple-phase product with a density of 2890 kg m^-3^; density was determined with a picnometer using distilled water as immersion liquid. The result is similar to the densities obtained by Cruells et al. [18], Patiño et al. [16] and Reyes et al. [19]. The resulting chemical composition (5.8% K, 20.4% Fe, 3.8% AsO_4_, 39.5% SO_4_, 30.3% H_3_O + OH + H_2_O) corresponds to a solid solution of synthetic jarosite with arsenic, with the following approximate formula:

$$\mathrm{KF}e_{3}{\left(SO_{4}\right)}_{1.82}\;{\left(\mathrm{AsO}\right)}_{0.18}\;{\left(\mathrm{OH}\right)}_{5.82}$$

The incorporation of arsenate into this compound is four times higher than the natrojarosite with arsenic obtained by Dutrizac et al. [8] and Reyes et al. [19].

**Figure 1:**
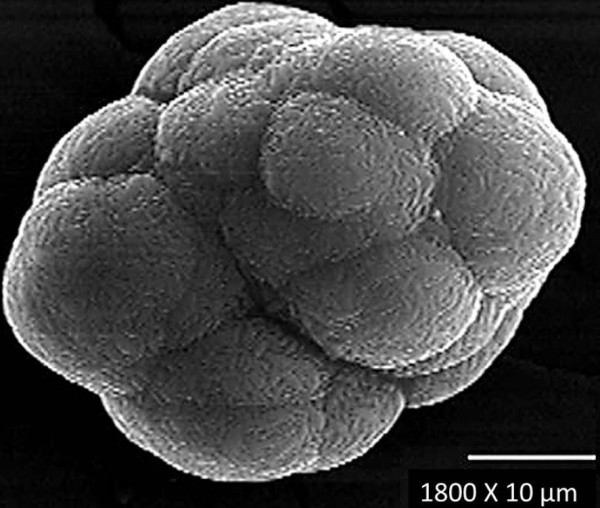
**Spherical aggregate of synthetic jarosite with arsenic (SEM, secondary electrons).** It has been reported that the potassium jarosite contains more arsenic in its structure than the sodium jarosite [8]. The spherical geometry is due to the fact that the particles were obtained by the precipitation technique with mechanical stirring; such geometry, as well as the compact structure are excellent parameters for heterogeneous kinetics studies.

**Figure 2:**
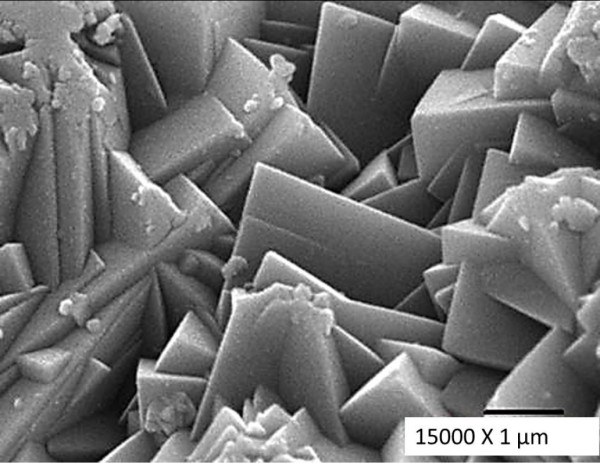
**SEM micrograph of the external surface of synthetic jarosite with arsenic (secondary electrons).** The spherical particles are made of rhombohedral crystals with sizes ranging from 2 to 8 μm; such crystals are soundly soldered in a compact structure.

The synthesis of jarosite with arsenic under the previously described conditions allows for the formation of spherical aggregates (Figure 1), which consist of rhombohedral crystals measuring between 2 and 8 μm (Figure 2) that form a compact texture. These are clearly favorable characteristics for kinetic modeling. Particle sizes of 38 μm, obtained by wet sieving, were used for the kinetic study in order to conduct a comparison with other decomposition studies of jarosite-type compounds, since it is the same diameter used in previous studies. Wet sieving is a technique that allows for good particle size separation.

## Experimental procedure

The alkaline decomposition experiment was carried out in a conventional thermostated glass kettle with magnetic stirring. The pH of the solution was constantly measured for each experiment with an Orion 3 Star pH-meter equipped with a Thermo Ross Ultra Sure Flow pH electrode, and the experiments were planned so that the reactant concentrations would be constantly adjusted. For the decomposition in NaOH and Ca(OH)_2_ media, both of ACS reagent grade, J. T. Baker, 0.4 g of synthetic jarosite with arsenic were used in an initial volume of 1 L. pH was kept constant in both media by adding small amounts of NaOH or Ca(OH)_2_ during the experiment. OH^-^ concentration was calculated according to the pH of the solution, and the ionization constant of water was calculated according to the working temperature [20].

The use of NaOH was to subject the synthetic jarosite with arsenic to extreme conditions of alkalinity. Besides, decomposition reactions in this media are free from interference kinetics (according to Patiño et al.) [14-17]. On the other hand, Ca(OH)_2_ is widely used in the industry as means to control pH, as well as in different environmental remediation processes (water treatment, soil treatment, etc.)

As previously noted, alkaline decomposition is characterized by the release of sulfate and potassium ions from the lattice, as well as by their quick diffusion towards the solution. In NaOH and Ca(OH)_2_ media, reaction progress was followed by taking 5 mL samples that were analyzed for potassium by atomic absorption spectrometry AAS (Perkin Elmer Analyst 200) and for sulfur by inductively coupled plasma ICP (Perkin Elmer Optima-3000XL).

The alterations due to sampling and addition of the reagent were corrected by mass balance. Of the three techniques (ICP, AAS, gravimetry), AAS was selected to follow the alkaline decomposition kinetics of the synthetic jarosite with arsenic. As already mentioned, several experiments were carried out to observe the evolution of the solids at different conversion values. These solids were characterized by chemical analyses, XRD, SEM-EDS and AAE.

For the stoichiometric study of the reaction, synthetic jarosite with arsenic samples were treated with NaOH and Ca(OH)_2_ for long periods of time. Later, sulfate and potassium in the solution were analyzed, and the residues were characterized by XRD, EDS and AAE.

## Results and discussion

### Decomposition of the synthetic jarosite with arsenic in NaOH and Ca(OH)_2_

#### Stoichiometry

**Figure 3:**
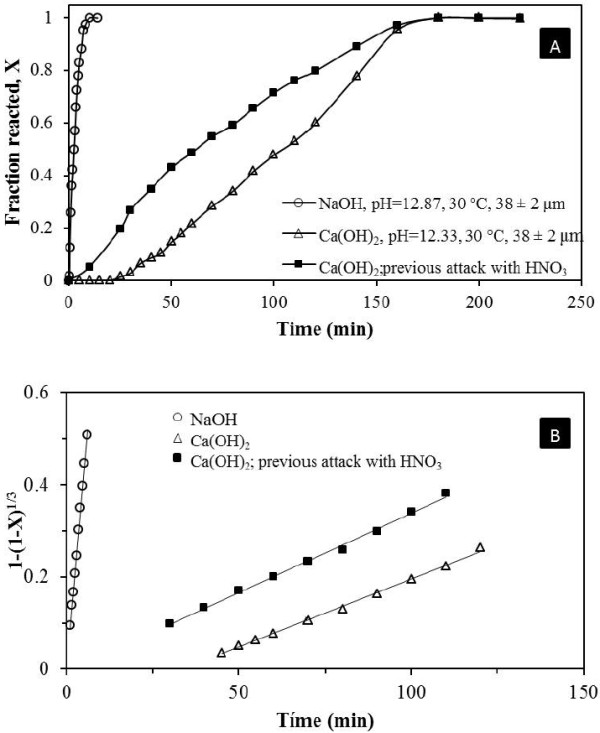
**(A) Decomposition curves and (B) model for chemical control.** The alkaline decomposition curves show an induction period, where the K and S percentages appear at negligible levels; during that period active points are formed until a reaction front is established. Afterwards, a progressive conversion period appears, where the K and S concentrations progressively increase until reaching stabilization, which indicates the reaction has concluded. The spherical particle model with decreasing core and chemical control was used to determine the rate experimental constant. This kinetic model has been applied to jarosites that do not contain arsenic, and it has been proved that it best describes the alkaline decomposition process [14-18].

**Figure 4:**
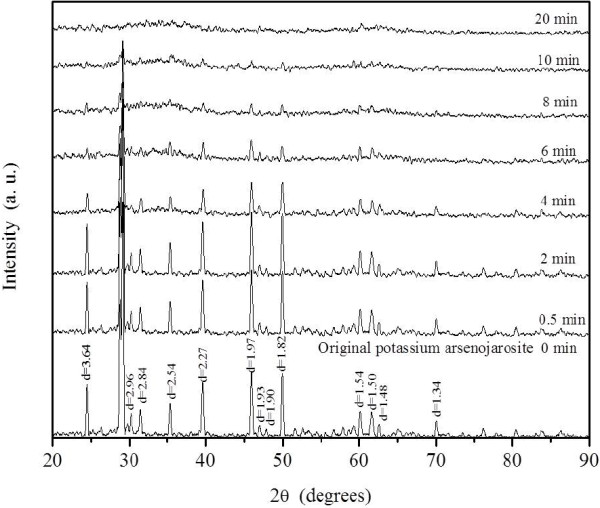
**Reaction products at different times.** (NaOH media, pH 12.87, 30°C and 38 ± 2 μm). As the reaction time passes, the intensity of the crystallographic planes decreases until disappearing; this results in an X-ray amorphous compound.

Results on the progress of sulfate and potassium during decomposition are shown in Figure 3A. Figure 4 shows the diffractograms of the residues corresponding to the data in Figure 3A (including times subsequent to total decomposition).

As previously stated, it can be observed that alkaline decomposition starts with an induction period (θ), during which the external appearance (color, morphology, etc.) of the synthetic jarosite with arsenic remains unaltered, and negligible traces of potassium ions were found in solution (Table 1). The diffractograms of the solids during this phase were also identical in position and intensity compared to those of the original synthetic jarosite with arsenic. The end of the induction period (θ) was identified by a change in the surface color of the solid, from yellow to orange.

**Table 1:** **K extraction at different time periods (NaOH, Ca(OH)**_**2 **_**and Ca(OH)**_**2 **_**attacked with HNO**_**3**_**)**

**MEDIA**					
**NaOH**		**Ca(OH)**_**2**_		**Ca(OH)**_**2 **_**attacked with HNO**_**3**_	
**Time**	**AAS**	**Time**	**AAS**	**Time**	**AAS**
	**K % Extraction**		**K % Extraction**		**K % Extraction**
0.0	0.0	0	0.00	0	0
0.3	1.9	5	0.00	10	5.3
0.5	12.7	10	0.00	25	19.7
1.0	25.9	15	0.00	30	26.8
1.5	36.0	20	0.00	40	34.9
2.0	42.1	25	1.4	50	43.0
2.5	50.1	30	2.9	60	48.7
3.0	57.0	35	6.5	70	54.9
3.5	65.9	40	8.6	80	59.2
4.0	72.4	45	10.3	90	65.6
4.5	78.0	50	14.6	100	71.4
5.0	83.1	55	17.8	110	76.2
6.0	88.1	60	21.6	120	79.8
7.0	95.3	70	28.3	140	89.1
8.0	>99.9	80	33.9	160	97.2
		90	41.5	180	>99.9
		100	47.7	200	>99.9
		110	53.0	220	>99.9
		120	60.0		
		140	77.8		
		160	95.5		
		180	>99.9		
		200	>99.9		
		220	>99.9		

**Figure 5:**
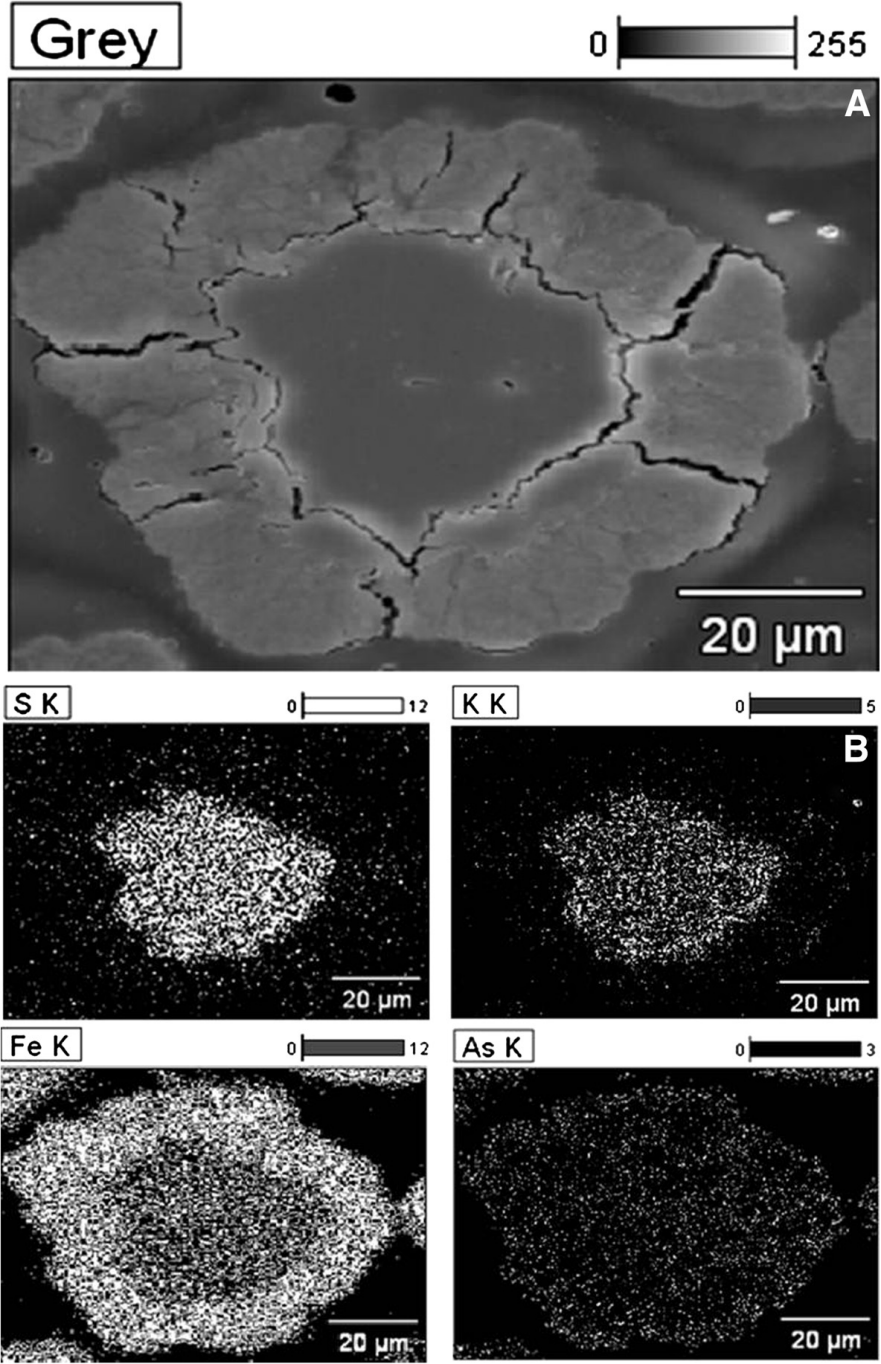
**(A) Partially reacted particle and (B) X-ray map.** Figure 5A shows a partially decomposed particle made of a gel halo, a reaction front and an unreacting core. Figure 5B shows the mapping performed on the partially decomposed particle. In the part showing the gel halo it can be observed how K and S have diffused from the particle into the solution, and are present only in the unreacting core. Fe and As are distributed throughout the particle. Previous studies have reported that the arsenic remains in the solids of the alkaline decomposition of arsenical natrojarosite [19].

In the conversion period, a reaction front was formed, (Figure 5A) the SO_4_^2-^ and K^+^ concentrations progressively increased (see Table 1), while simultaneously peak intensity of the synthetic jarosite with arsenic (ICDD PDF: 00-022-0827) decreased until XRD peaks disappeared (Figure 4). After [SO_4_^2-^] and [K^+^] reached stabilization, the reaction concluded. The solid residues were X-ray amorphous and did not evolve into crystal phases under the studied conditions of alkaline decomposition (as observed in Figure 4).

Accordingly, the stoichiometry of the process can be expressed by a reaction of the following type:

(1) $$\begin{array}{cc} \;\mathrm{KF}e_{3} & {\left(SO_{4}\right)}_{1.82}\;{\left(\mathrm{AsO}\right)}_{0.18}\;{\left(\mathrm{OH}\right)}_{5.82\left(S\right)}+3.180H_{\left(\mathrm{aq}\right)}^{-}\to K_{\left(\mathrm{aq}\right)}^{+}\\ & +1.82{\left(\mathrm{SO}\right)}_{4\left(\mathrm{aq}\right)}^{2-}\;3\mathrm{Fe}{\left(\mathrm{OH}\right)}_{3}\;1.18\;{\left(\mathrm{As}O_{4}\right)}_{\left(\mathrm{gel}\right)}^{3-} \end{array}$$

#### Decomposition curves

The alkaline decomposition kinetics was followed by AAS analysis of potassium. The first characteristic of the kinetic curves is the presence of an induction period (θ) that rises as temperature decreases and grows as [OH^-^] diminishes. This behavior has also been noticed in previous studies, e.g. synthetic argentian natrojarosite, synthetic argentian jarosite and synthetic natrojarosite with arsenic [16,18,19].

The solids underwent EDS analysis during the induction period, and there were no morphological changes or formation of a solid layer detectable at practical levels of resolution of the EDS technique (≈ 0.1 μm). This leads to the conclusion that the induction period is a superficial phenomenon at molecular thickness levels related to adsorption processes, whose detailed mechanism requires techniques that were not available for this study. At any rate, this could be the period during which active sites are created until the reaction front is established. It was proved that superficially attacking the synthetic jarosite with arsenic in HNO_3_ (a way to create defects or to remove submicron films) minimizes the induction period (θ). This was confirmed by conducting an experiment under the same conditions of decomposition in Ca(OH)_2_; results are shown in Figure 3. It can be noticed that the induction period diminishes, while keeping the same experimental rate constant. However, in this study, the induction period has not been eliminated, and its dependence on OH^-^ concentration, temperature and particle size have been determined. EDS testing of the solids after the induction period (θ) indicates the presence of a reaction front containing a non-reacting core of synthetic jarosite with arsenic and a gel halo of iron hydroxide with adsorbed arsenate (see Figure 5A). The presence of As in the solid residues was confirmed by AAE. It was observed that 100% of the arsenic remains in the residual solids in the decomposition experiments at 30°C. For long residence times (780 min) and high reaction temperatures (70°C), about 95% of the initial arsenic was retained in the solid, which reveals that at these conditions there is a slight diffusion of As toward the solution. Since the reaction occurs only at the interface, the kinetics has been described by separating the induction period from the conversion period.

Observation of a high number of cases indicates that the kinetic model of unreacted core gives a fairly accurate description of the decomposition of jarosite type compounds [14-19]. The controlling stage in a heterogeneous reaction is that which presents the highest resistance. In the spherical particle model with decreasing core two stages can be slow: matter transport through the ash layer (halo), or the chemical reaction at the interface between the unreacted core and the ash halo [21]. The potential control of the decomposition process by transport in the bulk of the solution during the conversion period was assessed by comparing the decomposition times obtained under diffusive control. The determined values indicate that the decomposition times are fast, which supports the conclusion that the process is not controlled by mass transfer [21-23].

Consequently, it was observed that ion diffusion through the decomposition gel was quick. This can be noticed in Figure 5B, where the presence of iron and arsenic is current over the particle, while potassium and sulfur are only visible in the core, which indicates that these two ions have diffused into solution. It can also be noticed that the distribution of Fe is not regular throughout the particle. Since only Fe and As are present in the halo, the proportion of iron is much higher than in the core, which remains unreacted.

Therefore, the model chosen for the conversion period was the spherical particle model with decreasing core and chemical control, which is expressed as [24]:

(2) 1-1-X13=Kexpt

(3) Kexp=VmKqCAnr0

Where *X* = reacted fraction; *K*_*q*_ = chemical constant; *C*_*A*_ = reactant concentration; *V*_*m*_ = molar volume of the solid; *r*_*0*_ = initial radius; *n* = reaction order.

Data are consistent with the selected model (Figure 3B). The values of the experimental rate constants *K*_*exp*_ were obtained by linear regression of equation (2) with a regression coefficient of r > 0.99. The values of the induction times (θ) were obtained by considering the intersections between the regression straight line and the time axis.

#### Dependence of the induction period

**Figure 6:**
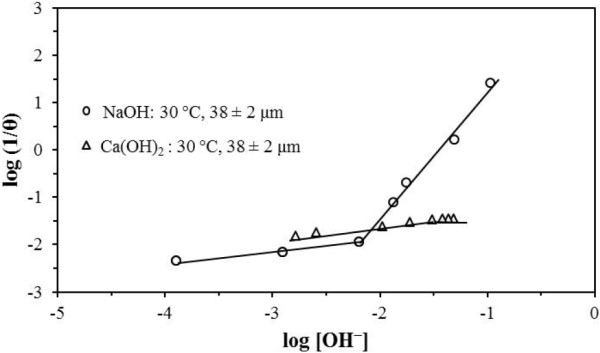
**Effect of [OH**^**-**^**] on the induction period, 30°C.** The duration of the induction period has a strong dependence on OH^-^; this is more evident in NaOH media, where the dependence of [OH^-^]^2.65^ was obtained. It was also observed that the duration of the induction period does not have a strong dependence on the OH^-^ concentration in Ca(OH)_2_, where the dependence was [OH^-^]^0.24^.

**Figure 7:**
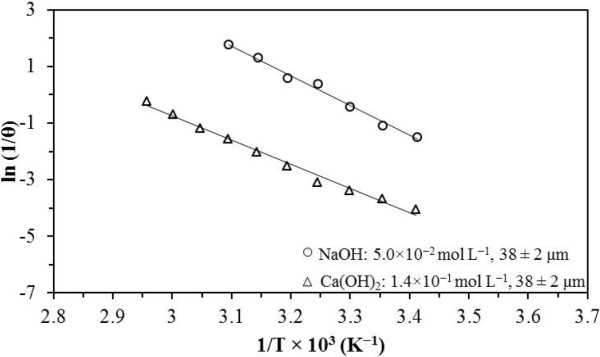
**Dependence of the induction period on temperature.** Temperature is a parameter that considerably affects the duration of the induction period, since the latter is proportional to temperature. The apparent activation energy values confirm that the chemical reaction is the stage that controls the process [21].

**Figure 8:**
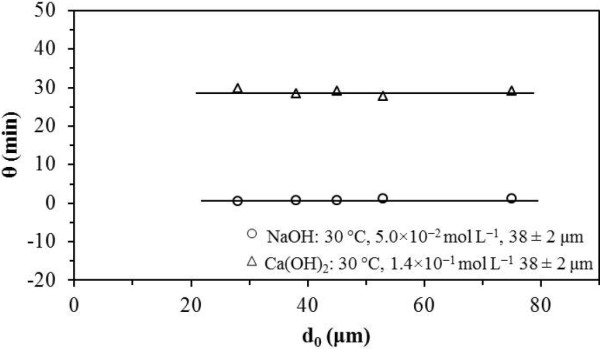
**Effect of the particle size on the induction period.** Both in NaOH and Ca(OH)_2_, the duration of the induction period is independent from the particle size.

In NaOH media for [OH^-^] 1.00 × 10^-4^ at 6.40 × 10^-3^ mol L^-1^, 1/θ was approximately proportional to OH^0.23^; for [OH^-^] > 6.40 × 10^-3^ mol L^-1^, 1/θ was proportional to OH^2.65^ (Figure 6). In Ca(OH)_2_ media for [OH^-^] 1.70 × 10^-3^ at 3.12 × 10^-2^ mol L^-1^, 1/θ was proportional to OH^0.24^; for [OH^-^] > 3.12 × 10^-2^ mol L^-1^, θ was independent from that concentration (Figure 6). The effect of temperature was exponential in both media. Figure 7 is a plot showing ln (1/θ) vs. 1/T. The values of energy dependence in NaOH and Ca(OH)_2_ media were approximately equal (84.6 and 88.2 kJ mol^-1^). Besides, in NaOH and Ca(OH)_2_, θ is independent from the particle size (Figure 8), which confirms that the activation process is a phenomenon occurring at the molecular scale.

#### Dependence of the conversion period

**Figure 9:**
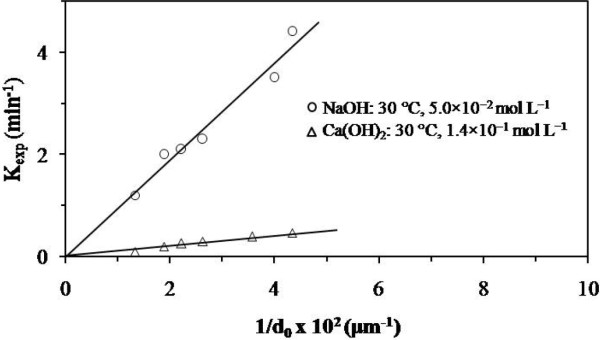
**Plot of the rate constants against the inverse of the initial particle diameter.** With small particle diameters the reaction rate increases, since the superficial area that reacts is smaller compared to the superficial area of larger particles.

Figure 9 shows the effect of particle size on the decomposition rate of the synthetic jarosite with arsenic. A plot of the experimental rate constant (determined at constant concentration and temperature) vs. the inverse of the particle diameter was linear and passes through the origin. This is consistent with the spherical particle model with decreasing core and chemical control (equation 2). Furthermore, this result rules out control by transport through the product layer; therefore, in this case the rate constant is inversely proportional to the square of the initial diameter (d_0_) [24].

**Figure 10:**
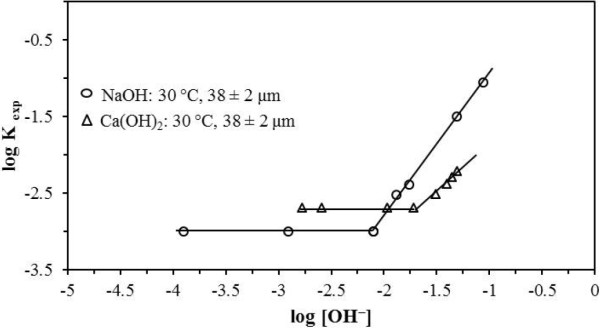
**Effect of [OH**^**-**^**] on the rate constants.** Concentration is another parameter that considerably affects the reaction rate, until a concentration is reached where the reaction rate no longer depends on the concentration; this occurs at lower concentrations in both media, yielding a reaction order equal to zero.

For the decomposition process in NaOH media for [OH^-^] 8.00 × 10^-3^ at 8.79 × 10^-2^ mol L^-1^, the reaction order (n) was 1.86, while for [OH^-^] lower than 8.00 × 10^-3^mol L^-1^, the reaction order (n) was zero (Figure 10). For the decomposition process in Ca(OH)_2_ media for [OH^-^] 1.90 × 10^-2^ at 4.49 × 10^-2^ mol L^-1^, the reaction order (n) was 1.15, while for [OH^-^] lower than 1.90 × 10^-2^, the reaction order (n) was zero (Figure 10).

**Figure 11:**
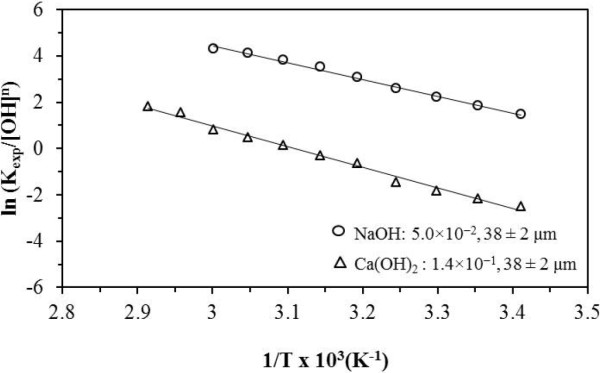
**Dependence of the conversion period on temperature.** Temperature considerably affects the reaction rate. In NaOH media, an apparent activation energy (Ea) of 60.3 kJ mol^-1^ was obtained, and in Ca(OH)_2_ the obtained apparent activation energy (Ea) was 74.4 kJ mol^-1^. These values are within the range of chemical control.

The temperature effect indicates an apparent activation energy (Ea) of 60.3 kJ mol^-1^ in NaOH media (Figure 11). The apparent activation energy (Ea) obtained in Ca(OH)_2_ media was 74.4 kJ mol^-1^. The apparent activation energy values are within the limits of chemical control.

According to Table 2, the reaction order (n) of synthetic jarosite with arsenic in NaOH media is 2.66 times higher than that of the synthetic natrojarosite with arsenic reported by Reyes et al. [19], indicating a strong dependence on OH^-^. In Ca(OH)_2_ media, the reaction orders (n) for both compounds are similar, but lower than those obtained in NaOH media. The table shows that the apparent activation energy (Ea) of the synthetic jarosite with arsenic in both alkaline media is higher than that obtained with the synthetic natrojarosite with arsenic reported by Reyes et al. [19].

**Table 2:** **Comparison of the reaction orders (n) and apparent activation energy (Ea) of the synthetic jarosite with arsenic and synthetic natrojarosite with arsenic in NaOH and Ca(OH)**_**2 **_**media respectively**

**Jarosite type**	**Media**	**[OH**^**-**^**]**	**Reaction order**	**Activation energy, Ea**
		**mol L**^**-1**^	**n**	**kJ mol**^**-1**^
Synthetic jarosite with arsenic ^a^	NaOH	>8.00 × 10^**-3**^	1.86	60.3
	Ca(OH)_2_	>1.90 × 10^**-2**^	1.15	74.4
Synthetic natrojarosite with arsenic ^b^	NaOH	>3.84 × 10^**-3**^	0.70	57.1
	Ca(OH)_2_	>2.21 × 10^**-2**^	1.51	48.6
Synthetic jarosite ^c^	NaOH	>2.70 × 10^**-2**^	0.6	43.0
	Ca(OH)_2_	>3.50 × 10^**-3**^	0.5	80.0

^a^ This work; ^b^ Reyes et al. [19]; ^c^ Cruells et al. [18].

According to these results, under the experimental conditions used for this work, the synthetic jarosite with arsenic has a higher energy dependence compared to the synthetic natrojarosite with arsenic; this difference in energy dependence becomes even more evident when compared to the values obtained in the alkaline decomposition of the synthetic jarosite without arsenic.

The elevated apparent activation energy of this compound is related to increased incorporation of arsenate (4 times higher compared to synthetic natrojarosite with arsenic [19]); this causes the lattice to expand [13], and as consequence, the amount of energy required for alkaline decomposition becomes much higher.

A comparison with other decomposition studies on synthetic jarosites without arsenic at similar conditions shows that the energy dependence is higher for ammonium jarosite [17] and argentian jarosite [25], and lower for plumbojarosite [15] and natrojarosite [16]. Regarding the concentration effect, there are no definite criteria by which a comparison can be made. This is because reagents diffuse in different ways on the particle surface. However, since the behavior of these compounds does not differ much, the natural compounds show a behavior similar to that of the studied synthetic compounds.

## Conclusions

1. The decomposition of the synthetic jarosite with arsenic in alkaline media presents an induction period, where there are no changes on the surface of the jarosite. It is followed by a conversion period, until total decomposition of the synthetic jarosite with arsenic is reached.

2. During the conversion period, sulfate and potassium ions diffused into the solution, remaining an X-ray amorphous iron hydroxide gel with adsorbed arsenate. In the alkaline decomposition in NaOH media for [OH^-^] 1.0 × 10^-4^ at 6.40 × 10^-3^ mol L^-1^, 1/θ was proportional to OH^0.23^. For [OH^-^] > 6.40 × 10^-3^ mol L^-1^, 1/θ was proportional to OH^2.65^, and apparent activation energy of 84.66 kJ mol^-1^ was obtained.

3. During the conversion period, for [OH^-^] > 8.00 × 10^-3^ mol L^-1^, the concentration order was proportional to [OH^-^]^1.86^. For [OH^-^] < 8.00 × 10^-3^ mol L^-1^ the effect is zero, and apparent activation energy of 60.3 kJ mol^-1^ was obtained. In the decomposition in Ca(OH)_2_ media for [OH^-^] 1.7 × 10^-3^ at 3.12 × 10^-2^ mol L^-1^, 1/θ was proportional to [OH^-^]^0.24^; for [OH^-^] > 3.12 × 10^-2^ mol L^-1^, θ was independent from OH^-^ concentration, and an apparent activation energy of 88.2 kJ mol^-1^ was obtained.

4. During the conversion period, for [OH^-^] > 1.90 × 10^-2^, the reaction order was 1.15. For [OH^-^] < 1.90 × 10^-2^ mol L^-1^the reaction order was zero and apparent activation energy of 74.4 kJ mol^-1^ was obtained. Under the studied conditions, the obtained activation energies are consistent with the spherical particle model with decreasing core and chemical control.

5. The energy dependence calculated for the synthetic jarosite with arsenic is higher than the calculated for the synthetic natrojarosite with arsenic under similar decomposition conditions; therefore it is more difficult for the alkaline decomposition of the synthetic jarosite with arsenic to start.

## Competing interests

The authors declare that they have no competing interests.

## Authors’ contributions

FP initiated the setup of experiments and ran the initial experiments and drafted the manuscript. MUF conducted most of the experiments and contributed to the discussion and manuscript outline. IAR conducted most of the experiments and contributed to the discussion and manuscript outline interpreted data. MR characterized and analyzed the decomposition solids. JH participated in experimental design and characterization of decomposition solids. IR assisted in analysis and interpretation of the data. JCJ characterized and analyzed the decomposition solids and contributed to the discussion and manuscript outline interpreted data. All authors read and approved the final manuscript.
